# A community resource for exploring and utilizing genetic diversity in the USDA pea single plant plus collection

**DOI:** 10.1038/hortres.2017.17

**Published:** 2017-04-26

**Authors:** William L. Holdsworth, Elodie Gazave, Peng Cheng, James R. Myers, Michael A. Gore, Clarice J. Coyne, Rebecca J. McGee, Michael Mazourek

**Affiliations:** 1 Plant Breeding and Genetics Section, School of Integrative Plant Science, Cornell University, Ithaca, NY 14853, USA; 2 Department of Crop and Soil Sciences, Washington State University, Pullman, WA 99164, USA; 3 Department of Horticulture, Oregon State University, Corvallis, OR 97331, USA; 4 US Department of Agriculture, Agricultural Research Service, Western Regional Plant Introduction Station, Pullman, WA 99164, USA; 5 US Department of Agriculture, Agricultural Research Service, Grain Legume Genetics and Physiology Research Unit, Pullman, WA 99164, USA

## Abstract

Globally, pea (*Pisum sativum *L.) is an important temperate legume crop for food, feed and fodder, and many breeding programs develop cultivars adapted to these end-uses. In order to assist pea development efforts, we assembled the USDA Pea Single Plant Plus Collection (PSPPC), which contains 431 *P. sativum *accessions with morphological, geographic and taxonomic diversity. The collection was characterized genetically in order to maximize its value for trait mapping and genomics-assisted breeding. To that end, we used genotyping-by-sequencing—a cost-effective method for *de novo *single-nucleotide polymorphism (SNP) marker discovery—to generate 66 591 high-quality SNPs. These data facilitated the identification of accessions divergent from mainstream breeding germplasm that could serve as sources of novel, favorable alleles. In particular, a group of accessions from Central Asia appear nearly as diverse as a sister species, *P. fulvum,* and subspecies, *P. sativum *subsp. *elatius*. PSPPC genotypes can be paired with new and existing phenotype data for trait mapping; as proof-of-concept, we localized Mendel’s *A *gene controlling flower color to its known position. We also used SNP data to define a smaller core collection of 108 accessions with similar levels of genetic diversity as the entire PSPPC, resulting in a smaller germplasm set for research screening and evaluation under limited resources. Taken together, the results presented in this study along with the release of a publicly available SNP data set comprise a valuable resource for supporting worldwide pea genetic improvement efforts.

## Introduction

Pea (*Pisum sativum* L.) is a globally important food, feed and cover crop in temperate environments. In 2014, green and dry peas had worldwide productions of 17.4 and 11.2 million tonnes, respectively, making pea the fourth largest legume crop after soybean, groundnut and common bean.^1^ The nutritive benefits associated with pea have prompted the USDA to specify ‘beans and peas’ as one of five distinct vegetable subgroups recommended for regular consumption (http://www.choosemyplate.gov/), a decision supported by dietary studies showing that consumption of these legumes is correlated with higher intakes of fiber, protein and an array of vitamins and minerals.^2,3^ Comprising ~25% protein, pea seed can be used as a protein source in many animal feeds.^4,5^ In addition, as a cool-season and non-transgenic substitute for soybean, pea has the potential for organic systems and in short-season areas where local feed sources are prioritized but where soybean production is limited.^6–8^ As a rotation or cover crop, in association with *Rhizobium* bacteria, pea can fix atmospheric nitrogen at levels sufficient to produce subsequent vegetable and cereal crops with reduced application of additional fertilizers.^9,10^

Breeding efforts to develop pea cultivars have largely resulted in the partitioning of pea germplasm into distinct groups primarily differentiated by end-use and market type,^11,12^ for example, snap and snow peas with edible pods for the fresh and frozen markets, shelling peas for processing and field peas for use as a whole food, for animal feed or fractionated as a component in processed food. This sort of partitioning, along with subsequent crossing of elite lines, has been associated with decreased levels of genetic diversity in a number of crop species.^13,14^ The genetic bottleneck associated with pea improvement has not been as severe as in some crops and when collectively considering landraces and accessions from across all breeding programs, much diversity has been retained.^11,15,16^ This is presumably because alleles critical for different end-uses and growing environments have been maintained in their respective breeding programs.^11,15^ However, the genetic diversity within individual breeding programs can be restrictively narrow.^17,18^. In addition, non-elite and wild germplasm pools most likely contain novel, favorable alleles not represented in these programs.^14,19^

In order to maintain novel alleles in non-elite germplasm, many pea germplasm collections have been assembled. Sixteen collections housed in Europe, Asia and North America contain over 1000 accessions.^20^ From these collections, core collections have been identified that consist of more manageable numbers of accessions, often ~10% of the original collections.^21^ Consisting of 504 accessions, the USDA core collection was assembled based on geography and flower color, and represented ~18% of all USDA pea accessions at the time of construction.^22,23^ To facilitate genetic analysis of the collection, homozygous accessions were derived by single-seed descent from a subset of the core to form the ‘Pea Single Plant’ (PSP) collection.^24^ The under-representation of genetically distinct Chinese accessions^12^ within the PSP collection led us to modify and augment this collection to form the USDA PSP Plus Collection (PSPPC), first reported here. The PSPPC includes 344 accessions from the PSP collection,^23–25^ accessions from the Chinese core collection and field, snap and snow peas from US public pea-breeding programs. Taxonomically, the PSPPC contains accessions from the primary cultivated subspecies, *Pisum sativum* subsp. *sativum*, as well as from each of the two currently accepted wild subspecies, *P. sativum* subsp. *elatius* and *P. sativum* subsp. *abyssinicum*.^26^ These wild subspecies can be distinguished from the cultivated subspecies by a set of morphological characteristics, for example, early flowering and strongly serrated leaflets in *P. sativum* subsp. *abyssinicum* and deshiscent pods in *P. sativum* subsp. *elatius*, as well as a reciprocal translocation that is characteristic of *P. sativum* subsp. *abyssinicum* accessions and many but not all of *P. sativum* subsp. *elatius* accessions.^26^ Geographically, PSPPC accessions are diverse, with robust representation from the center of domestication, that is, the Near East and Mediterranean,^26^ and other centers of diversity, including Central Asia and Ethiopia.^27^

The objective of this research was to use genotyping-by-sequencing (GBS), a reduced-representation library sequencing approach, to generate a publicly available, high-density marker data set for the PSPPC to maximize its value for trait mapping and genomics-assisted breeding. Reduced-representation library sequencing has been used in a number of crop plants to discover and simultaneously score numerous single-nucleotide polymorphism (SNP) markers across the entire genome.^28,29^ In pea, reduced-representation library sequencing was recently used to construct a genetic linkage map that included 64 263 SNP markers for a historically important ‘Baccara’ x PI 180693 RIL population.^30^ Here, we generated 66 591 high-quality SNPs for the 431 samples of the PSPPC. To demonstrate the utility of our SNP marker data set for varying end-use applications, we identified accessions genetically distant from cultivated germplasm as potential new sources of diversity for breeding programs. We also mapped a previously cloned gene that regulates flower color in close proximity to its known position, showing that our high-density marker data set represents a resource that can be rapidly used to allow breeders to connect genotypes to phenotypes at a higher resolution. Finally, we constructed a high utility, smaller core collection of 108 accessions that captures 97% of the SNP allelic diversity found in the PSPPC.

## Materials and methods

### Plant material

**Figure 1:**
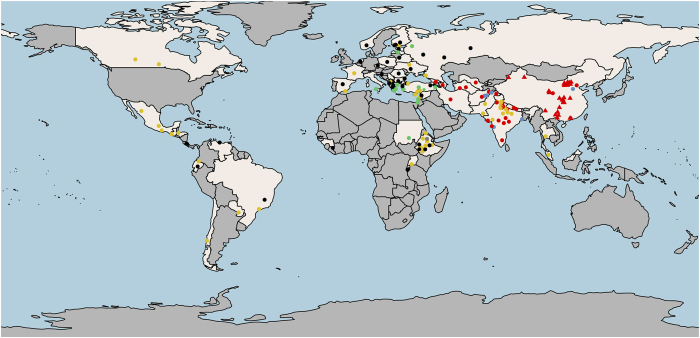
Map of collected accessions of the PSPPC. Of 431 *P. sativum* accessions studied, 238 were collected from 52 countries. The remaining accessions were donated to the collection from an unknown origin or developed by plant breeders. Circles indicate accessions in the original PSP collection and triangles indicate accessions from the Chinese core collection. Diamonds indicate remaining accessions. Colors correspond to genetic groupings discussed later herein: *P. sativum* subsp. *elatius* (green), *P. sativum* subsp. *abyssinicum* (gray), *P. sativum* subsp. *sativum*—Primary (gold), *P. sativum*—Central Asia (dark blue) and *P. sativum* subsp. *sativum*—non-Mediterranean Asia (red).

A total of 431 *P. sativum* accessions are included in the PSPPC, with descriptor information provided in Supplementary Table S1. Where applicable and available, this information includes the following: USDA accession numbers, status as ‘Collected,’ ‘Developed’ (through breeding), or ‘Donated’ (collection origin unknown), availability according to the USDA Germplasm Resources Information Network (GRIN), membership in the original PSP collection, subspecies and passport information including country of origin and latitude and longitude coordinates. For accessions with location names or country origins only, GPS Visualizer (www.gpsvisualizer.com) was used to assign position coordinates using Google Maps Geocoding API. The snap and snow pea accessions are from Oregon State University and the field pea accessions are from the USDA Agricultural Research Service Grain Legume Genetics and Physiology Research Unit at Washington State University. The ‘rworldmap’ package in R was used to plot accessions that were collected (Figure 1).^31^

Twenty-five accessions of *P. fulvum* were sequenced as an outgroup for diversity analyses. *P. fulvum,* found only in the Middle East,^26^ is the only other widely accepted species within the *Pisum* genus, and is distinguished from *P. sativum* by crossing barriers, DNA polymorphism and morphological features, for example, dehiscent pods and seed dormancy.^14,26,32^ These accessions are listed in Supplementary Table S2.

### GBS of the PSPPC

The PSPPC accessions were sequenced using GBS. Leaf tissue was harvested from one individual seedling of each accession grown in a greenhouse, and total genomic DNA was extracted in a plate format using the DNeasy 96 Plant Kit (Qiagen, Valencia, CA, USA). GBS libraries of pooled samples were prepared by the Genomic Diversity Facility at Cornell University as previously described.^29^ The restriction enzyme *Ape*KI was used to digest the total genomic DNA samples. This methylation-sensitive restriction enzyme preferentially cleaves within undermethylated gene-rich regions of plant genomes, thus allowing targeted sequencing of the low-copy, genic fraction in the pea genome—a large genome that primarily consists of highly repetitive DNA.^33^ The GBS libraries were sequenced using a HiSeq 2500 Illumina Sequencing System (Illumina Inc., San Diego, CA, USA).

SNPs were identified from 100 base-pair sequence reads using the TASSEL 3.0 Universal Network Enabled Analysis Kit (UNEAK) and Stacks v1.19, two SNP-calling pipelines that do not require a reference genome for read alignment.^34,35^ Non-reference pipelines were used because of a preliminary analysis that found that reference-based SNP-calling with alignment to the closest sequenced *Pisum* relative, *Medicago truncatula*, yielded fewer than half of the number of SNPs as the non-reference pipelines. This is presumably due to significant divergence between *Pisum* and *Medicago* since their split ~25 million years ago.^36^ To call SNPs, each of the pipelines (UNEAK and Stacks) were run twice: once on the PSPPC alone and once including *P. fulvum* accessions (data set hereafter referred to as PSPPC+*P. fulvum*). For the Stacks pipeline, reads with intact barcodes from fastq files were demultiplexed, stripped of barcodes and truncated to 80 base pairs (bp) with the process_radtags function (-t 80 -e apeKI -i fastq). SNPs were called using the denovo_map.pl function using the following described parameters (-m 4 -M 1 -N 3 -n 1 -t -X ustacks:--max_locus_stacks 2). At least four identical reads (m) from each individual were grouped into ‘stacks’. Highly repetitive reads were removed (t). Loci for each individual were assembled by allowing one mismatch (M) between a maximum of two stacks (-X ustacks:--max_locus_stacks). Secondary reads containing up to three mismatches (N) were added to primary loci and a consensus sequence with the identified SNP was called. A catalog of loci from all individuals was created with one mismatch (n) allowed between loci, and SNPs were called by matching individual loci against the catalog loci. For the UNEAK pipeline, reads from fastq files with intact barcodes and no ‘N’s in the first 64 bp were demultiplexed, stripped of barcodes and truncated to 64 bp using the UFastqToTagCountPlugin function (-e ApeKI). A ‘tag’ was defined as the consensus sequence of identical reads from a single individual. Using the UMergeTaxaTagCountPlugin function, only tags present in at least five accessions (-c 5) were retained in the analysis. With the UTagCountToTagPairPlugin function and an error tolerance rate (-e 0.03) of 0.03, a network filter was used to identify reciprocal tag pairs that comprised putative loci. Sequence reads from accessions that were sampled as biological replicates were combined and processed as a single accession.

Custom Perl scripts were used to call marker genotypes and to filter loci. For each accession, marker genotypes at a locus were considered ‘homozygous’ if fewer than 5% of the total sequence reads for that locus were the less-sequenced ‘alternate’ allele, ‘missing’ if 5–10% of the total reads were the alternate allele and ‘heterozygous’ if 10% or more of the total reads were the alternate allele. In addition, SNP markers were excluded from the data set when they met at least one of the following conditions: their minor allele frequency was lower than 0.01, their accession call rate (that is, the fraction of taxa that had a non-missing genotype) was lower than 0.2 or their heterozygosity rate was greater than 0.25. This latter threshold on heterozygosity was chosen because it is above the level of heterozygosity expected for any locus in a mostly inbred collection, but sufficient to filter out paralogous SNP loci. In Stacks, for sequences with more than one SNP, only the first SNP in the sequence passing all filtering criteria was retained. The consensus sequences of retained SNP markers from Stacks was aligned to the consensus sequences of retained SNP markers from UNEAK using the BLASTN algorithm in the BLAST 2.2.28 stand-alone package with an E-value cutoff of 0.01.^37,38^ A final data set for analysis was assembled using the union of SNPs from the UNEAK and Stacks pipelines. Individual genotypes at shared SNPs were those called by UNEAK.

### Identifying diversity with the potential for pea breeding

To identify sources of novel alleles for cultivar development, we calculated the number of alleles represented in certain genetic groups but not in the Agricultural Research Service and Oregon State University breeding program germplasm. The PSPPC+*P. fulvum* accessions were divided into groups based on specific and subspecific taxonomic classification (for example, *P. fulvum* and *P. sativum* subsp. *elatius*) or in the case of the main cultivated subspecies, *P. sativum* subsp. *sativum,* from two previous studies that defined population structure for an overlapping subset of accessions.^24,25^ In these previous studies, two subpopulation groups for *P. sativum* subsp. *sativum* were defined by the program STRUCTURE. We assigned PSPPC accessions to either the primary cultivated group, which we termed ‘*P. sativum* subsp. *sativum—*Primary’ or the smaller group with phenotypic attributes resembling that of undomesticated accessions and from Central Asia, which we termed ‘*P. sativum*—Central Asia’. For each accession, group membership was assigned if STRUCTURE values were equal to or greater than 0.85 for the same group in both studies^25^ (Cheng *et al.;*^24^ unpublished data, Supplementary Table 1). Only three accessions from *P. sativum* subsp. *abyssinicum* were included in the PSPPC, and so this group was excluded from the diversity analysis because the sample size was too small to draw meaningful conclusions. Also excluded were accessions not included, reportedly admixed or placed in different genetic groups by Cheng *et al.*^24^ and Kwon *et al.*^25^ A custom python script was used to compare the number of unique alleles in each of the genetic groups with all germplasm and with breeding lines from Oregon State University and Agricultural Research Service. To account for the difference in sample size and missing data between these groups, all groups were downsampled so that each group had a score of 7.59±0.5, where score was calculated as the sum of (1-proportion missing data) for randomly chosen individuals until the threshold 7.59 was reached, which was the total score of the group with the least amount of data, *P. fulvum*. The number of unique SNPs was calculated on the downsampled groups. This procedure was repeated 100 times and the number of unique SNPs in each group was obtained by averaging the number of unique SNPs over the 100 iterations. Genetic diversity of collected and developed accessions was visualized using principal component analysis (PCA). The ppca function from the pcaMethods package in R^39^ was used to calculate 10 principal components for both the PSPPC and the PSPPC+*P. fulvum* data sets (Supplementary Tables 1 and 2).

### GWAS of flower color

**Figure 2:**
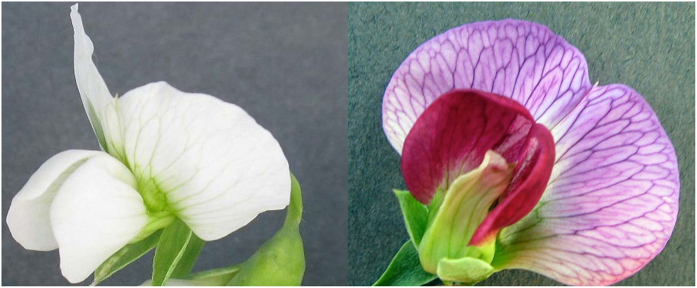
Examples of flower color phenotypes for GWAS. PI 156720 (left) has a white flower and PI 195020 (right) has a pigmented flower.

To demonstrate the utility of GBS-derived SNPs for dissecting the genetic basis of phenotypic variation in *Pisum*, flower color controlled by the ‘*A*’ gene—a previously molecularly characterized locus^40^—was studied. PSPPC flower color phenotypes were either classified as ‘pigmented’ or ‘white’ (Figure 2). For PSPPC accessions from the PSP collection, phenotypes were downloaded from the GRIN website using the ‘flower color’ and ‘PSP’ descriptors. For PSP accessions without flower color phenotype data, phenotypes were assigned using photographs and data from the original PI accessions from which the inbred PSP accessions were derived. In instances where data from two or more studies were in contradiction or unavailable, the phenotype value was recorded as ‘NA’. For breeding lines, phenotypes were reported by breeders James Myers and Rebecca McGee from Oregon State University and Agricultural Research Service, respectively. Phenotype data are provided in Supplementary Table 1. The PSPPC union data set that included all SNPs from both UNEAK and Stacks pipelines at a minimum sample call rate of 20% and minor allele frequency of 1% was used as the genotype data. Statistical tests of association between flower color and SNP markers were conducted using a mixed linear model implemented within the Genome Association and Prediction Integrated Tool (GAPIT) package in R.^41,42^ To control for population structure and relatedness, the mixed linear model included principal components and a kinship matrix^43^ that were calculated using the data set of 66 591 SNPs in GAPIT. Only the first principal component was included to control for population structure as determined by the Bayesian information criterion.^44^ A Bonferroni correction^45^ was used to control for the multiple testing problem by adjusting the alpha value from *α*=0.05 to *α*=(0.05/66 591) where 66 591 is the number of statistical tests conducted (that is, number of tested SNPs). Therefore, statistical significance of a SNP–trait association was set at 7.5e^−^^7^.

Given the genomic collinearity between *M. truncatula* and *P. sativum* in the region of the *A* locus,^40^ pea sequence reads containing SNPs statistically significant at a Bonferroni correction of 5% were aligned via BLASTN to the J. Craig Venter Institute *M. truncatula* genome 4.0 using an *E*-value cutoff of 1e^−5^ and blastn-short default parameters.^46^ To evaluate the proximity of these SNPs to the *A* locus, the 11 892 *A* locus nucleotide sequence (complete coding sequence) from the pea accession PI 269818 (GU132941.1) was also aligned to *M. truncatula* via BLASTN using the same parameters.

### Construction of a PSPPC mini-core collection

Accessions in the USDA pea core collections were selected based on geographic and morphological diversity in order to preserve underlying levels of genetic diversity. With high-density marker data, genetic diversity can be evaluated directly, and an optimal core identified based on a number of thresholds including total number of alleles or genetic distance between individuals.^47^ The software CoreHunter 2.0 was used to determine a minimum set of individuals from the PSPPC from among those available in GRIN that retained at least 95% of the alleles present in the full PSPPC data set.^47,48^ To this end, CoreHunter was run iteratively with the sample intensity parameter decreasing from 0.95 to 0.05 by 0.05 for each iteration with the following parameters remaining constant: runtime: 10 min and CV (allele coverage)=1. For each output, minor allele frequency was determined using a custom python script. A principal component analysis was conducted on the resultant PSPPC mini-core using the same methods as described for the PSPPC and PSPPC+*P. fulvum* data sets.

## Results

### GBS of the PSPPC

A total of 66 591 SNPs were called in the 431 accessions of the PSPPC data set. When 25 *P. fulvum* accessions were included, the same pipeline and filters called a total of 67 400 SNPs in the 456 accessions of the PSPPC+*P. fulvum* data set (Table 1). On average, these SNPs had a non-missing genotype in at least 53% of the samples (Table 1). When considering only the SNPs with a minimum read depth of five reads across all samples, 16 675 and 18 097 SNPs were called in the PSPPC and PSPPC+*P. fulvum* collections, respectively. These SNPs supported by higher coverage were genotyped in more than 80% of the samples (20% or less missing taxa for each SNP; Table 1).

### Identifying diversity with potential for pea breeding

**Figure 3:**
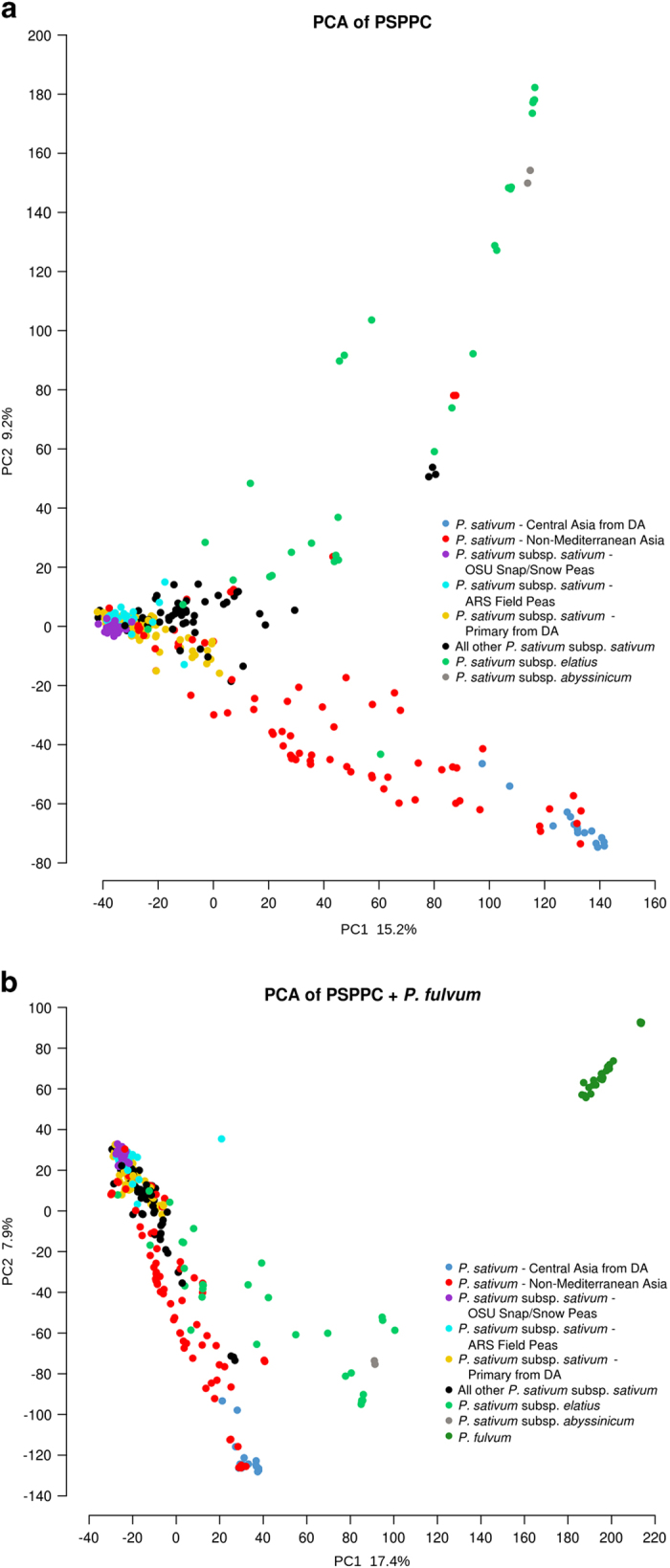
(**a**.) Principal Components 1 and 2 for Collected and Developed Accessions of the PSPPC and (**b**) PSPPC+*P. fulvum* (bottom). (**a**) The *P. sativum* subsp. *sativum*—Primary genetic group (gold) largely clustered with the breeding germplasm (cyan, purple). Peas from subspecies *P. sativum* subsp. *elatius* (light green) and the *P. sativum*—Central Asia group (dark blue) are distinct from cultivated germplasm. Most of the peas that form a gradient between the *P. sativum* subsp. *sativum*—Primary and *P. sativum*—Central Asia genetic groups are from Asia outside of the Mediterranean region (red). (**b**) The wild species *P. fulvum* (dark green) is the most differentiated group, clustering on its own apart from all other *P. sativum* groups. The accessions from DA (‘Diversity Analysis’) refer to *P. sativum* accessions in either of the two groups defined by Cheng *et al.*^24^ and Kwon *et al.*^25^ and used to find unique alleles compared with breeding germplasm.^24,25^

We performed two analyses to characterize the genetic diversity within accessions of the PSPPC and PSPPC+*P. fulvum* collections. First, we used a PCA to represent the genetic variation among accessions. Only collected and developed accessions are depicted for ease of visualization (Figure 3). Second, we counted the number of alleles for each of the non-breeding germplasm groups that were not present in the breeding material, and refer to these as unique alleles (Table 2). The PCA showed that the *P. fulvum, P*. *sativum* subsp. *elatius* and *P. sativum* -Central Asia groups were the most differentiated groups from the breeding germplasm (Figure 3a). These three groups also contained between two to four times more unique alleles than the geographically diverse, but genetically homogeneous *P. sativum* subsp. *sativum*—Primary group (Table 2). This result was consistent with the PCA that showed the *P. sativum* subsp. *sativum*—Primary group clustering with breeding germplasm (Figure 3a). The PCA also revealed a gradient of differentiation within *P. sativum* subsp. *sativum*, running from the most cultivated germplasm on one end to the *P. sativum*—Central Asia group on the other end. Accessions between these groups had a strong geographical component, with the majority originating from Asia outside of the Mediterranean region (Figure 3a). With few exceptions, *P. sativum* subsp. *sativum* was genetically distinct from *P. sativum* subsp. *elatius* and *P. sativum* subsp. *abyssinicum* (Figure 3a), and all *P. sativum* formed a genetically distinct group from the wild species *P. fulvum* (Figure 3b).

### Genome-wide association study of flower color

A genome-wide association study (GWAS) of flower color was conducted with 66 591 SNP markers in the GAPIT software package.^41,42^ Twenty-five SNP markers were significantly associated with flower color at the 5% Bonferroni-corrected threshold (Supplementary Table S3). Of these 25 markers, nine aligned to the *M. truncatula* genome sequence, and all of them localized within a 10.2 Mb interval on chromosome one (Supplementary Table S4). Importantly, this chromosome is known to contain the *A* locus homolog.^40^ The relative position of the *A* locus homolog was verified by the alignment of the *A* nucleotide sequence (complete coding sequence) from *P. sativum* accession PI 269818 to *M. truncatula* (Supplementary Table S4). Ten of twelve distinct sequence fragments from the *P. sativum A* sequence uniquely aligned to *M. truncatula*, delineating an 8-kb region contained within the GWAS-defined 10.2 Mb interval on chromosome one of *M. truncatula*. Furthermore, one of these sequence fragments had an alignment length of 942 bp and an *e*-value of 2e^−137^ (Supplementary Table S4). Of the SNPs identified to significantly associate with flower color in our GWAS, TP100211 (*P *value 1.16e^−08^) aligned 1244 bp from the nearest blastn-anchored, *P. sativum A* sequence fragment (Supplementary Table S4).

### Construction of a USDA mini-core collection

**Figure 4:**
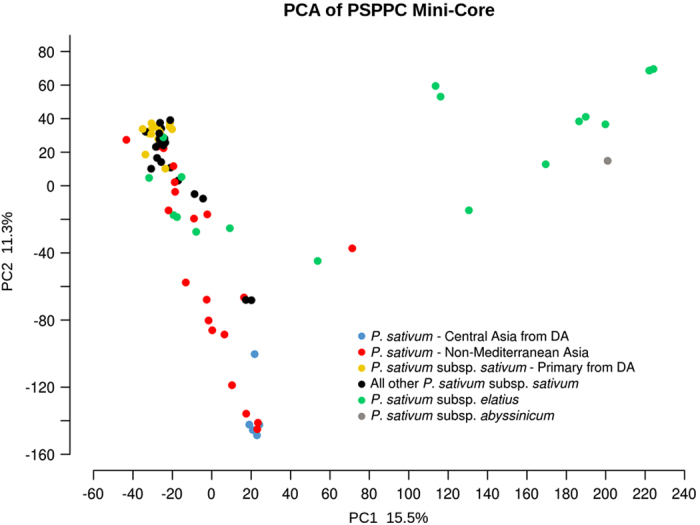
Principal components 1 and 2 for collected and developed *P. sativum* accessions in the PSPPC mini-core. The structure of the plot resembles the PCA of the full collection (Figure 3a), indicating robust representation of genetic groups in the PSPPC mini-core. Peas from subspecies *P. sativum* subsp. *elatius* (light green) and the *P. sativum*—Central Asia genetic group (dark blue) are distinct from cultivated germplasm. Most of the peas that form a gradient between the *P. sativum*—Primary and *P. sativum*—Central Asia genetic groups are from Asia outside of the Mediterranean region (red). The accessions from the ‘DA’ (Diversity Analysis) refer to *P. sativum* accessions in either of the two groups defined by Cheng *et al.*^24^ and Kwon *et al.*^25^ and used to find unique alleles compared with breeding germplasm.^24,25^

Using only the accessions from the PSPPC that are publicly available in GRIN, a PSPPC mini-core of 108 individuals was constructed that sampled 97.4% of the 133 182 alleles in the PSPPC. In addition, 97.0% of all 66 591 markers have minor allele frequencies equal to or greater than 0.01, the original threshold for the PSPPC SNP data set. The PCA structure of the PSPPC core closely resembles the original PSPPC (Figure 4).

## Discussion

A GBS procedure was used to score 66 591 SNP markers across 431 diverse *P. sativum* accessions of the PSPPC, representing one of the largest marker data sets in pea to date. Without the current availability of a *P. sativum* reference genome sequence, we used two non-reference-genome-enabled SNP-calling pipelines, UNEAK and Stacks. Pipelines with differing methodologies for SNP calling can yield distinct sets of SNPs, to the extent that in some cases, less than 50% of SNPs are shared.^49^ The advantages of each of multiple pipelines can be leveraged to identify a larger number of SNPs for downstream analyses. For instance, UNEAK is better suited to call genotypes from low-coverage loci, whereas Stacks is better suited to call genotypes from loci characterized by more than one SNP, that is, haplotypes.

The PSPPC SNP data set is publicly available and has utility for identifying germplasm with potential to increase genetic diversity in pea-breeding programs. In particular, peas from Central Asia, historically termed ‘Afghanistan’ types after the predominant country of origin,^50^ cluster distinctly from breeding accessions and most other *P. sativum* accessions (Figure 3). In this respect, our data agree with many past studies.^12,14,25,51–54^ Afghanistan-type accessions within European collections have been described as being nearly as distinct from cultivated pea as is *P. fulvum*.^14,51,55^ Our PCA results lend support to the classification of the *P. sativum*—Central Asia group as a separate subspecies, genetically differentiated from each of the widely accepted subspecies *P. sativum* subsp. *elatius*, *P. sativum* subsp. *abyssinicum* and *P. sativum* subsp. *sativum*. Future phylogenetic studies may elucidate whether a subspecies from this group is more rigorous than the current classification of *P. sativum* subsp. *elatius,* which is primarily based on a small number of morphological traits including dehiscent pods, and is increasingly considered a genetically paraphyletic group.^14,56–58^

For randomly chosen subsets of taxonomic and genetic groups standardized to account for missing data, the Central Asia group contained more SNPs absent from the breeding germplasm than other *P. sativum* subsp. *sativum*—Primary accessions, and nearly as many new alleles as *P. sativum* subsp. *elatius* and *P. fulvum*. In addition, the Central Asian accessions contained over 6000 alleles not represented in any of the other groups of accessions sampled, including *P. fulvum*. However, the number of alleles reported for *P. fulvum* may be artificially low for genomic regions significantly diverged from *P. sativum*; these would not be captured by the reference-independent SNP-calling pipelines. The genetic diversity of Central Asian accessions is mirrored by their morphological diversity, which prompted Vavilov^59^ and Govorov^60^ to describe Central Asia as a primary center of origin for pea,^59^ in addition to other centers including the Near East.^60,61^ In our Central Asia group from the diversity analysis, peas were from just five countries (Afghanistan, China, India, Nepal and Pakistan), while accessions in the *P. sativum* subsp. sativum—Primary group were from 37 countries spanning six continents (Supplementary Table S1). Alleles in the Central Asia group and from other genetically similar Asian accessions could contribute favorably to traits such as: disease resistance, cold hardiness and early maturation in addition to non-obvious traits for which positive alleles are masked in unfavorable genetic backgrounds.^19,60,62^ Wild (sub)species may contain similar alleles with utility for breeding programs,^63,64^ although crossing barriers such as chromosomal rearrangements between wild species and cultivated material can inhibit the transfer of these alleles.^32,65^ On the contrary, no crossing barriers are known to exist between the Afghanistan types and other cultivated *P. sativum*, making this group a valuable source of alleles for improvement of breeding germplasm.^66^

Phenotype data for the USDA pea collections have enabled breeders to identify useful germplasm for breeding programs, but the dense molecular marker data needed to identify robust marker–trait associations have been lacking. Previous genetic mapping efforts for important physiological and agronomic traits, such as seed mineral concentration, nematode resistance, days to flowering and biomass production, have identified some marker–trait associations, but low marker densities have prevented the detection of tight linkage between markers and candidate genes.^24,25^ The PSPPC data set is available as a ‘GWAS-ready’ public resource. Derived primarily from the PSP collection, the PSPPC is highly inbred. By using inbred accessions for phenotyping, researchers can remove within-accession genetic variance common in genetically heterogeneous USDA accessions that are maintained in the way that they are received. Given the high level of linkage disequilibrium in pea,^11,24,67^ a marker data set consisting of tens of thousands of SNPs should be sufficient in most association studies to tag important major genes, given amenable minor allele frequencies and sufficient population sizes. As proof-of-concept, we genetically pinpointed the previously identified *A* gene with SNP markers generated in this study and flower color phenotypes available from GRIN. All of the most significant *P. sativum* SNPs aligned to the same *M. truncatula* genomic interval that contained the *A* gene homolog. In addition, one of the significant SNPs from our GWAS, TP100211, was located less than 1.5 kb from the *A* locus.

Numerous other Mendelian genes and major-effect quantitative trait loci control agronomic traits of importance for pea-breeding programs, but are yet to be fine-mapped and cloned. These include genes for resistance to powdery mildew, *Fusarium* wilt, ascochyta blight and pea rust, in addition to stringlessness, snap pods and cold tolerance.^68–74^ With the appropriate phenotype data, PSPPC SNPs can be used to map these and other important traits. In addition, as *P. sativum* genome sequences become available, the raw GBS sequences can be used to call additional SNPs with reference genome-based pipelines and thereby help improve statistical power for mapping relatively smaller effect genes controlling polygenic traits.^75^

The PSPPC SNP data set facilitated the formation of a mini-core collection of 108 accessions that retained nearly all of the diversity of the larger PSPPC (Supplementary Table S1). The PSPPC mini-core can be considered a foundation on which to expand for phylogenetic and trait-mapping studies. This core may also be useful for germplasm curators, who, under resource constraints, could prioritize regeneration and distribution of a smaller number of accessions.

## Conclusion

A high-density SNP data set is now available for the PSPPC, a public resource with high utility for pea improvement. Genotype information will complement phenotype data already available to allow pea curators, breeders and geneticists to explore and utilize genetic diversity in pea.

### Data availability

For the PSPPC and PSPPC+*P. fulvum* SNP data sets, hapmap and vcf files as well as corresponding FASTA sequences are available on the USDA Ag Data Commons—DOI: 10.15482/USDA.ADC/1347137 (https://data.nal.usda.gov/dataset/data-community-resource-exploring-and-utilizing-genetic-diversity-usda-pea-single-plant-plus), the Cool Season Food Legume database (https://www.coolseasonfoodlegume.org/PubDatasets) and on GRIN-GLOBAL (https://npgsweb.ars-grin.gov/gringlobal/method.aspx?id=495893). SNP names that begin with a ‘TP’ are derived from the TASSEL SNP-calling pipeline, while SNP names that include ‘_’ are derived from the Stacks SNP-calling pipeline. SNPs for each of the PSPPC and PSPPC+*P. fulvum* groups were called independently; therefore, any SNP name that is shared between these groups should NOT be assumed to refer to the same locus. All raw sequencing data are available through the National Center for Biotechnology Information (NCBI) Sequence Read Archive (SRA) with BioProject number: PRJNA379298 and BioSample numbers: SAMN06604244-SAMN06604699 (http://www.ncbi.nlm.nih.gov/bioproject/379298) listed in Supplementary Tables S1 and S2. For each accession, raw reads were demultiplexed using the GBSX demultiplexer function, with no mismatches allowed for the barcode or enzyme sequences.^76^

## Figures and Tables

**Table 1:** Total number of SNP markers at different read depths

	*PSPPC*	*PSPPC+P. fulvum*
*All filtered markers*		
SNP number	66 591	67 400
Average read depth	4.1	4.4
Average percent missing taxa/SNP	47	47
		
*Filtered markers with read depth ⩾5*		
SNP number	16 675	18 097
Average read depth	11.7	12.2
Average percent missing taxa/SNP	18	20

Abbreviations: PSPPC, Pea Single Plant Plus Collection; SNP, single-nucleotide polymorphism.

For both germplasm collections, the numbers represent the UNEAK–Stacks union data set with loci called in at least 20% of individuals and having a minor allele frequency *⩾*1%.

**Table 2:** Summary of unique alleles for breeding programs

	*All others*	*ARS field peas*	*OSU snap peas*	*All breeding germplasms*
*P. fulvum*	8180	14 894	17 378	13 605
*P. sativum* subsp. *elatius*	7988	21 791	26 572	18 191
*P. sativum—*Central Asia	6079	16 045	19 426	13 357
*P. sativum* subsp. s*ativum*—Primary	2044	9938	14 180	6368

Abbreviations: ARS, Agricultural Research Service; OSU, Oregon State University.

Each count represents the average number of alleles found in the group on the left but not found in the group across the top. Comparisons were performed between random subgroups standardized for missing data (see Materials and Methods).
