# Supplementary material for: A community resource for exploring and utilizing genetic diversity in the USDA pea single plant plus collection
Source: Hortic Res. 2017 Apr 26;4:17017–. doi: 10.1038/hortres.2017.17 (PMC5405346; doi:10.1038/hortres.2017.17)
Supplement: Supplementary Table Legends [file hortres201717-s1.docx]

Table S1- Descriptor information for the PSPPC.

Table S2- Descriptor information for the PSPPC + *P. fulvum* set of accessions.

Table S3- SNPs significantly associated with flower color controlled by the *A* locus in pea at the 5% Bonferroni-corrected threshold.

Table S4- Alignment of the most significant markers detected in GWAS along with the *A* locus sequence from *P. sativum* accession PI 269818 to *M. truncatula*. The SNP marker TP100211 aligned less than 1.5 kb from the nearest *A*-gene fragment on *M. truncatula* chromosome one.
